# Supplementary material for: Structural mechanism of protein recognition by the FW domain of autophagy receptor Nbr1
Source: Nat Commun. 2022 Jun 25;13:3650. doi: 10.1038/s41467-022-31439-5 (PMC9233695; doi:10.1038/s41467-022-31439-5)
Supplement: Supplementary file 1 — Supplementary Information [file 41467_2022_31439_MOESM1_ESM.pdf]

Supplementary Information for

Structural mechanism of protein recognition by the FW domain of autophagy receptor Nbr1

Jianxiu Zhang, Ying-Ying Wang, Zhao-Qian Pan, Yulu Li, Jianhua Sui, Li-Lin Du and Keqiong Ye

Supplementary Figure 1-5
Supplementary Table 1-3

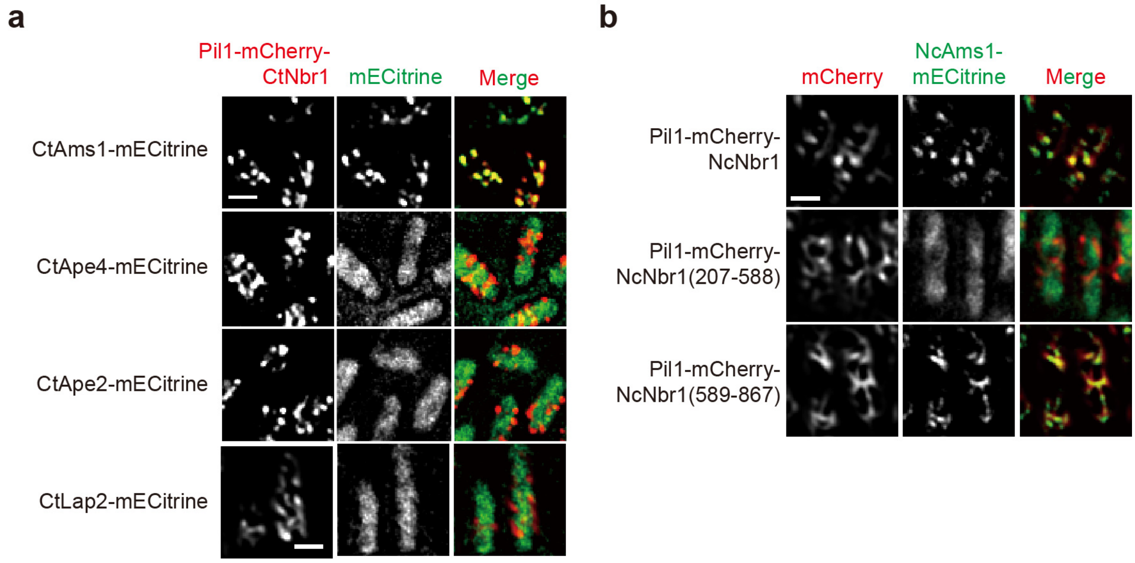

Supplementary Figure 1. Pil1 co-tethering assay of Nbr1 and its candidate cargo proteins.

a CtNbr1 was fused to Pil1-mCherry and co-expressed with CtAms1, CtApe4, CtApe2 and CtLap2 fused to mECitrine in *S. pombe*. Cells were imaged by fluorescence microscopy. Bar, 3 μm. **b** *Neurospora crassa* (Nc) Nbr1 was fused to Pil1-mCherry and co-expressed with NcAms1 fused to mECitrine in *S. pombe*. Data shown are representative of two independent experiments with similar results. Source data are provided as a Source Data file.

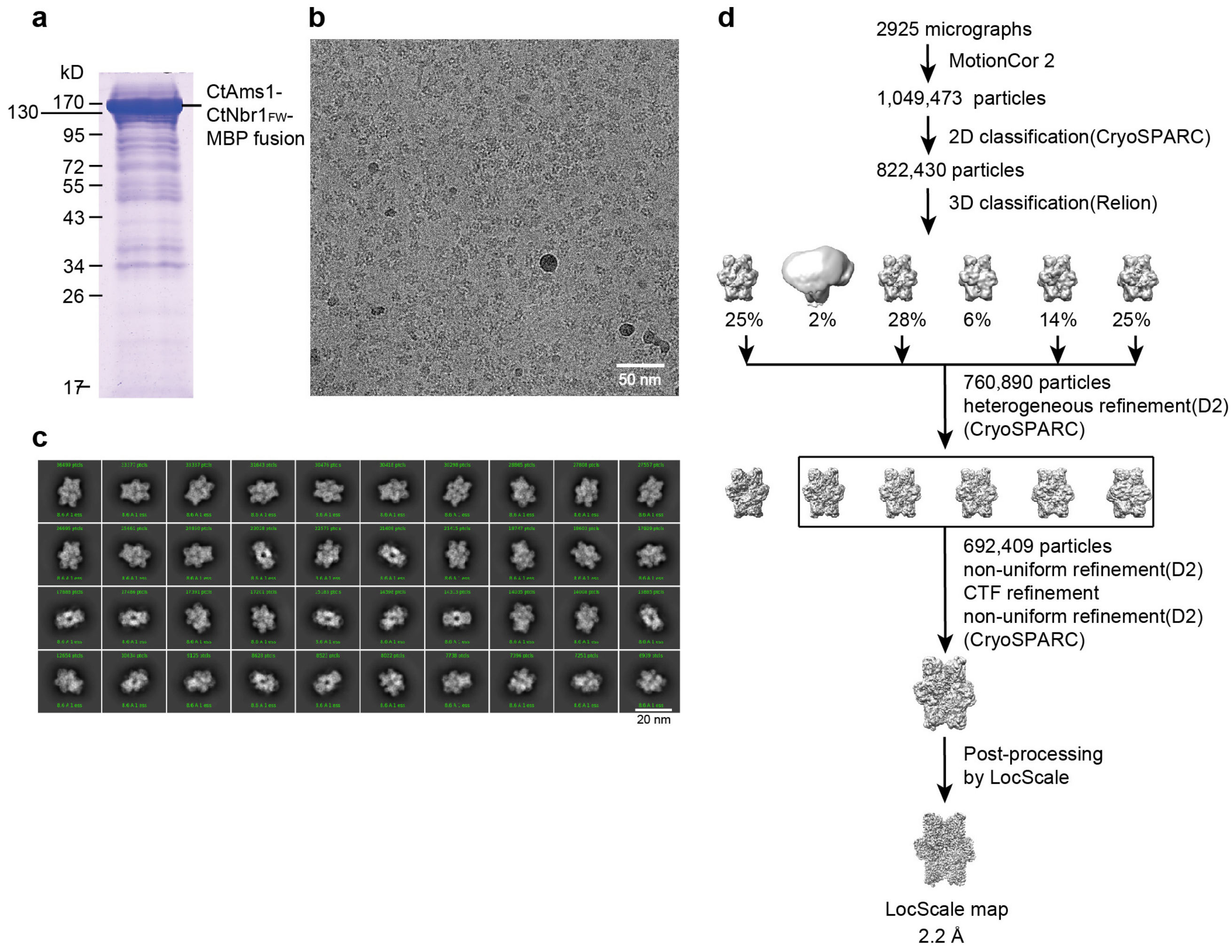

Supplementary Figure 2. Cryo-EM analysis of CtAms1 fused to the FW domain of CtNbr1.

a An SDS-PAGE image of the purified fusion protein. Source data are provided as a Source Data file. **b** A typical cryo-EM micrograph. Bar, 50 nm. **c** Representative 2D class averages. Bar, 20 nm. **d** Flowchart of data processing.

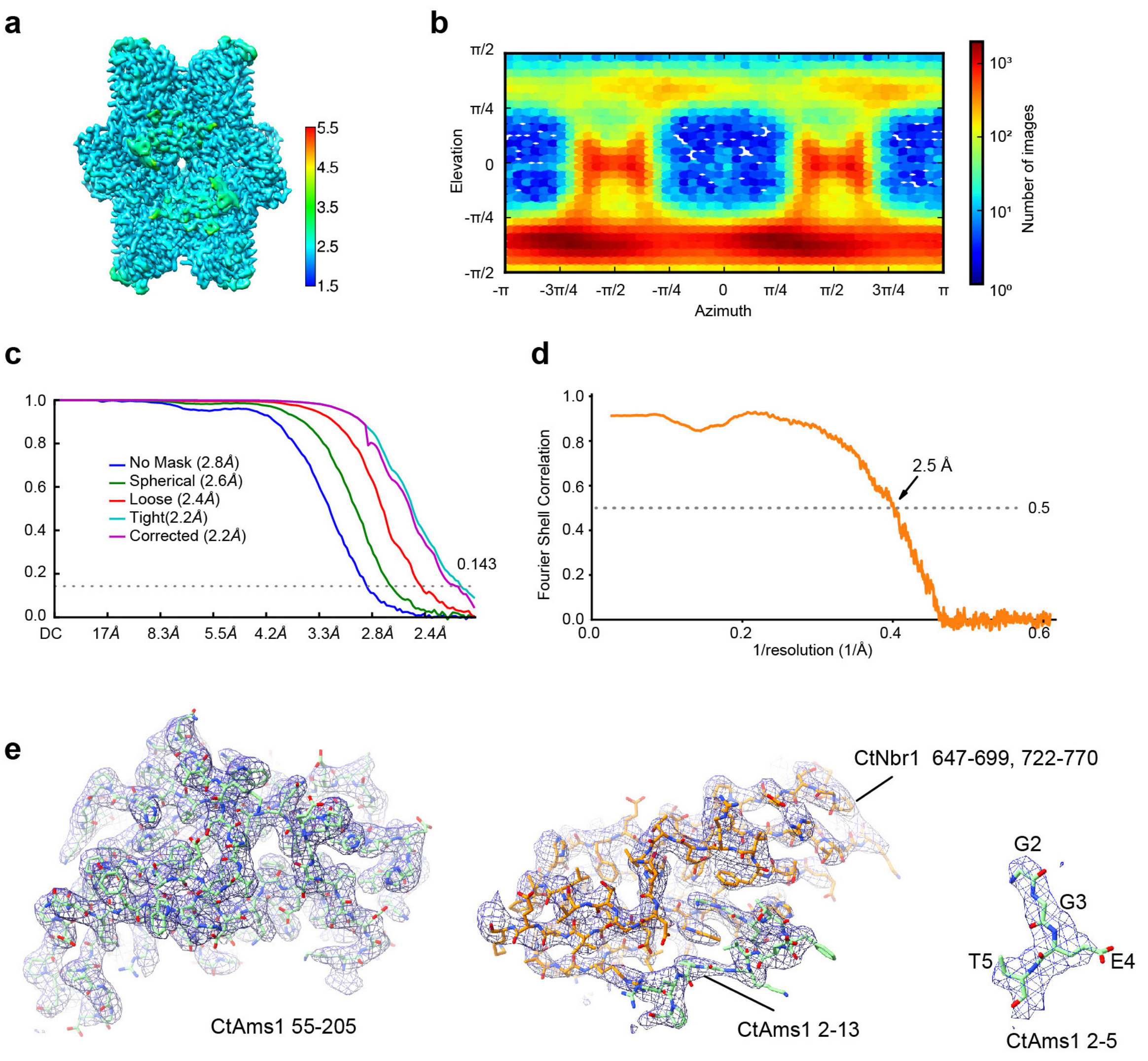

Supplementary Figure 3. Quality of the cryo-EM map of the CtAms1-FW complex.

a Cryo-EM density map is colored by local resolution. The map is sharpened by LocScale. **b** Angular distribution of particles. **c** Gold-standard FSC curves from cryoSPARC. **d** Map-to-model FSC curve between the refined model and the sharpened map. **e** Representative densities from the LocScale map are fitted with the final structural models.

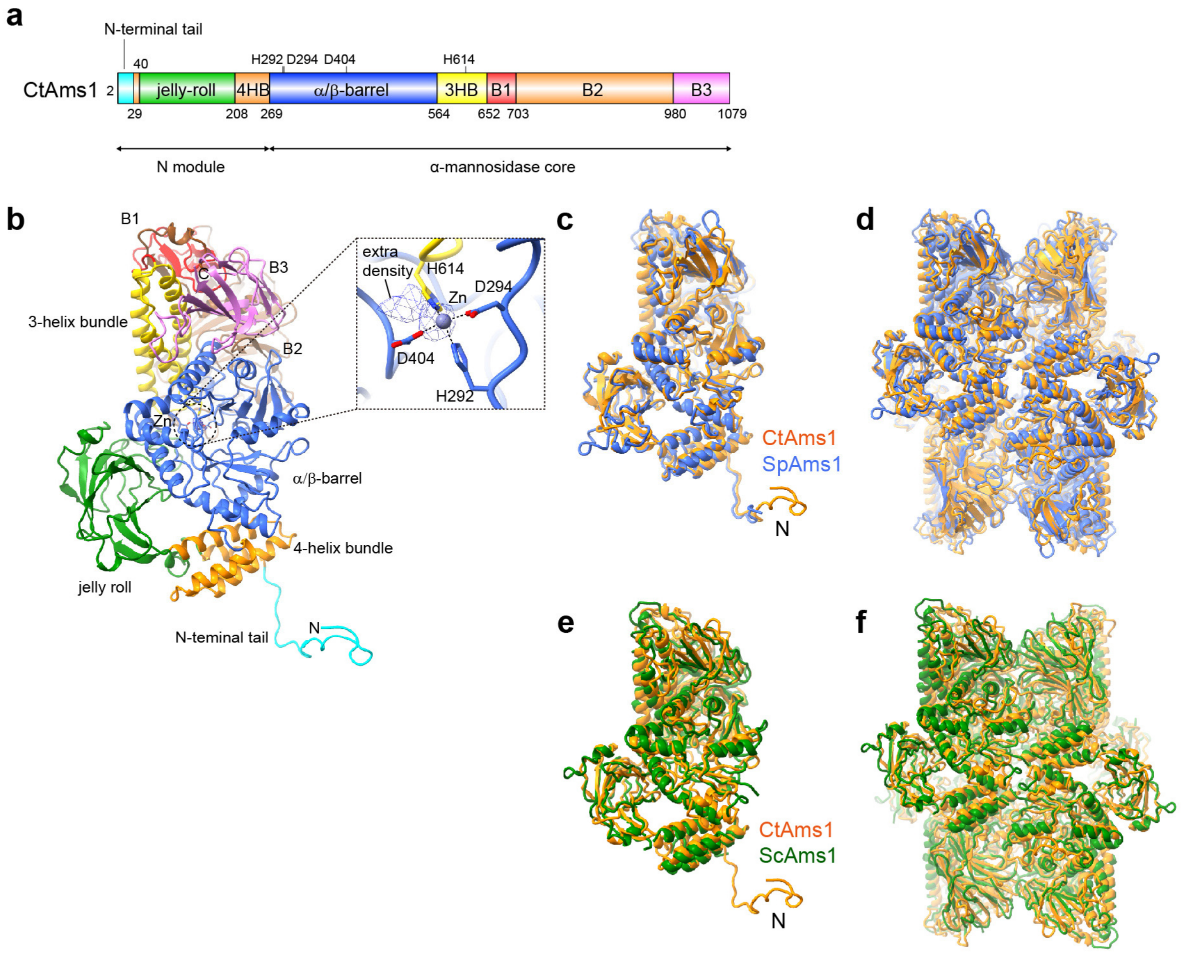

Supplementary Figure 4. Structure of CtAms1.

a Domain diagram of CtAms1. The N-terminal tail, a jelly-roll domain, a 4-helix bundle (4HB), an α/β-barrel, a 3-helix bundle (3HB), and the B1, B2, and B3 β-sheet domains are color-coded. The zinc-binding residues are marked. **b** Ribbon representation of the CtAms1 monomer structure. Domains are colored as in A. The insert shows the binding of one zinc ion (sphere) in the active site, with coordinating bonds shown as dashed lines. Cryo-EM density is displayed for the Zn ion and an unmodeled molecule. **c** Superposition of the monomer structures of CtAms1 (orange) and SpAms1 (blue, PDB code 6LZ1, 3.2 Å). The N-termini are labeled. The RMSD is 0.74 Å over 898 Cα atom pairs. **d** Superposition of the tetramer structures of CtAms1 and SpAms1. **e** Superposition of the monomer structures of CtAms1 (orange) and ScAms1 (green, PDB code 5JM0, 6.3 Å). The RMSD is 1.29 Å over 445 Cα atom pairs. **f** Superposition of the tetramer structures of CtAms1 and ScAms1.

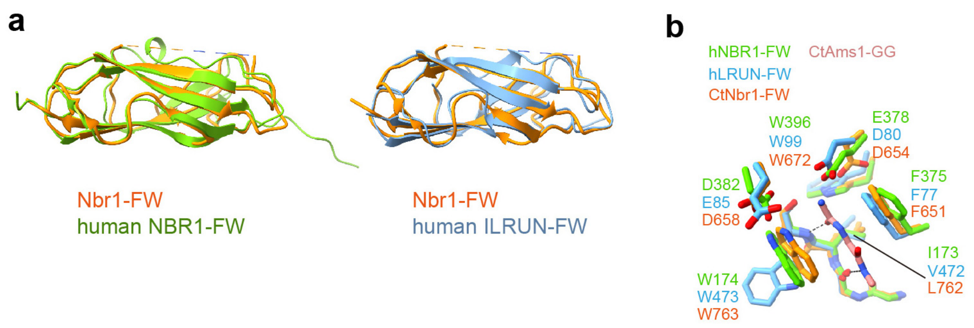

Supplementary Figure 5. Structural comparison of FW domains.

a Structural alignment of the FW domain of CtNbr1 with the FW domain from human NBR1(PDB code 4OLE) and human ILRUN (6VHI). The RMSD is 0.90 Å over 90 Cα atom pairs and 0.93 Å over 75 Cα atom pairs respectively. **b** Superposition of the ligand binding pockets of the FW domain of CtNbr1, human NBR1 and human ILRUN.

Supplementary Table 1. Yeast strains

Name	Mating type	Genotype
LD328	h+	*leu1-32 his3-D1*
LD259	h+	*leu1-32 his3-D1 ura4-D18*
DY49502	h-	*ura4-D18 ars1::pDUAL-P41nmt1-CtAms1-mECitrine(ura4+) leu1-32::pDUAL-P41nmt1-Pil1-mCherry-CtNbr1(leu1+)*
DY49503	h-	*ura4-D18 ars1::pDUAL-P41nmt1-CtAms1-mECitrine(ura4+) leu1-32::pDUAL-P41nmt1-Pil1-mCherry-CtNbr1(635-775)(leu1+)*
DY49204	h-	*ura4-D18 ars1::pDUAL-P41nmt1-CtAms1-mECitrine(ura4+) leu1-32::pDUAL-P41nmt1-Pil1-mCherry-CtNbr1(222-588)(leu1+)*
DY49205	h-	*ura4-D18 ars1::pDUAL-P41nmt1-CtAms1-mECitrine(ura4+) leu1-32::pDUAL-P41nmt1-Pil1-mCherry-CtNbr1(589-883)(leu1+)*
DY49504	h-	*ura4-D18 ars1::pDUAL-P41nmt1-CtApe1-mECitrine(ura4+) leu1-32::pDUAL-P41nmt1-Pil1-mCherry-CtNbr1(leu1+)*
DY49206	h-	*ura4-D18 ars1::pDUAL-P41nmt1-CtApe1-mECitrine(ura4+) leu1-32::pDUAL-P41nmt1-Pil1-mCherry-CtNbr1(635-775)(leu1+)*
DY49207	h-	*ura4-D18 ars1::pDUAL-P41nmt1-CtApe1-mECitrine(ura4+) leu1-32::pDUAL-P41nmt1-Pil1-mCherry-CtNbr1(222-588)(leu1+)*
DY49208	h-	*ura4-D18 ars1::pDUAL-P41nmt1-CtApe1-mECitrine(ura4+) leu1-32::pDUAL-P41nmt1-Pil1-mCherry-CtNbr1(589-883)(leu1+)*
DY49050	h+	*his3-D1 leu1-32::pDUAL-Pnmt1-CtAms1-30aa-CtNbr1-FW-13aa-MBP-2x3C-GFP(leu1+) ams1Δ::kanMX*
DY49049	h+	*his3-D1 ura4-D18 ars1::pDUAL-P41nmt1-Pil1-mCherry-CtNbr1-(635-775)(ura4+) leu1-32::pDUAL-P41nmt1-CtAms1-mECitrine(leu1+)*
DY49051	h+	*his3-D1 ura4-D18 ars1::pDUAL-P41nmt1-Pil1-mCherry-CtNbr1-(635-775)Y739A(ura4+) leu1-32::pDUAL-P41nmt1-CtAms1-mECitrine(leu1+)*
DY49052	h+	*his3-D1 ura4-D18 ars1::pDUAL-P41nmt1-Pil1-mCherry-CtNbr1-(635-775)D654A(ura4+) leu1-32::pDUAL-P41nmt1-CtAms1-mECitrine(leu1+)*
DY49053	h+	*his3-D1 ura4-D18 ars1::pDUAL-P41nmt1-Pil1-mCherry-CtNbr1-(635-775)D658A(ura4+) leu1-32::pDUAL-P41nmt1-CtAms1-mECitrine(leu1+)*
DY49054	h+	*his3-D1 ura4-D18 ars1::pDUAL-P41nmt1-Pil1-mCherry-CtNbr1-(635-775)R743A(ura4+) leu1-32::pDUAL-P41nmt1-CtAms1-mECitrine(leu1+)*
DY49055	h+	*his3-D1 ura4-D18 ars1::pDUAL-P41nmt1-Pil1-mCherry-CtNbr1-(635-775)W763A(ura4+) leu1-32::pDUAL-P41nmt1-CtAms1-mECitrine(leu1+)*
DY49056	h+	*his3-D1 ura4-D18 ars1::pDUAL-P41nmt1-Pil1-mCherry-CtNbr1-(635-775)(ura4+)*

		leu1-32::pDUAL-P41nmt1-CtAms1-G2A-mECitrine(leu1+)
DY49057	h+	*his3-D1 ura4-D18 leu1-32::pDUAL-P41nmt1-Pil1-mCherry-CtNbr1-(635-775)(leu1+) ars1::pDUAL-P41nmt1-CtAms1-G3A-mECitrine(ura4+)*
DY49058	h+	*his3-D1 ura4-D18 ars1::pDUAL-P41nmt1-Pil1-mCherry-CtNbr1-(635-775)(ura4+) leu1-32::pDUAL-P41nmt1-CtAms1-(G2-T5)Δ-mECitrine(leu1+)*
DY49059	h+	*his3-D1 ura4-D18 leu1-32::pDUAL-P41nmt1-Pil1-mCherry-CtNbr1-(635-775)(leu1+) ars1::pDUAL-P41nmt1-CtAms1-N14R-mECitrine(ura4+)*
DY49060	h+	*his3-D1 ura4-D18 ars1::pDUAL-P41nmt1-Pil1-mCherry-CtNbr1-(635-775)D694A(ura4+) leu1-32::pDUAL-P41nmt1-CtAms1-mECitrine(leu1+)*
DY49061	h+	*his3-D1 ura4-D18 ars1::pDUAL-P41nmt1-Pil1-mCherry-CtNbr1-(635-775)Y695A(ura4+) leu1-32::pDUAL-P41nmt1-CtAms1-mECitrine(leu1+)*
DY49062	h+	*his3-D1 ura4-D18 ars1::pDUAL-P41nmt1-Pil1-mCherry-CtNbr1-(635-775)R761A(ura4+) leu1-32::pDUAL-P41nmt1-CtAms1-mECitrine(leu1+)*
DY49063	h+	*his3-D1 leu1-32::pDUAL-P41nmt1-Pil1-mCherry-CtNbr1-(635-775)(leu1+) ars1::pDUAL-P41nmt1-CtAms1-R539A-mECitrine(ura4+)*
DY49505		*his3-D1 ura4-D18 ars1::pDUAL-P41nmt1-Pil1-mCherry-CtNbr1-(635-775)F651A(ura4+) leu1-32::pDUAL-P41nmt1-CtAms1-mECitrine(leu1+)*
DY49212	h-	*ura4-D18 ars1::pDUAL-P41nmt1-Pil1-mCherry-CtNbr1(635-775)D654E(ura4+) leu1-32::pDUAL-P41nmt1-CtAms1-mECitrine(leu1+)*
DY49213	h-	*ura4-D18 ars1::pDUAL-P41nmt1-Pil1-mCherry-CtNbr1(635-775)D654E(ura4+) leu1-32::pDUAL-P41nmt1-CtAms1-G2A-mECitrine(leu1+)*
DY49214	h-	*ura4-D18 ars1::pDUAL-P41nmt1-Pil1-mCherry-CtNbr1(635-775)D654E(ura4+) leu1-32::pDUAL-P41nmt1-CtAms1-G3A-mECitrine(leu1+)*
DY49043	h+	*his3-D1 ura4-D18 ars1::pDUAL-P41nmt1-Pil1-mCherry-CtNbr1(ura4+) leu1-32::pDUAL-P41nmt1-CtAms1-mECitrine(leu1+)*
DY49115	h+	*his3-D1 ura4-D18 ars1::pDUAL-P41nmt1-Pil1-mCherry-CtNbr1(ura4+) leu1-32::pDUAL-P41nmt1-CtApe2-mECitrine(leu1+)*
DY49116	h+	*his3-D1 ura4-D18 ars1::pDUAL-P41nmt1-Pil1-mCherry-CtNbr1(ura4+) leu1-32::pDUAL-P41nmt1-CtApe4-mECitrine(leu1+)*
DY49215	h-	*ura4-D18 ars1::pDUAL-P41nmt1-CtLap2-mECitrine(ura4+) leu1-32::pDUAL-P41nmt1-mCherry-Pil1-CtNbr1(leu1+)*
DY49209	h-	*ura4-D18 ars1::pDUAL-P41nmt1-NcAms1-mECitrine(ura4+) leu1-32::pDUAL-P41nmt1-Pil1-mCherry-NcNbr1(leu1+)*
DY49210	h-	*ura4-D18 ars1::pDUAL-P41nmt1-NcAms1-mECitrine(ura4+) leu1-32::pDUAL-P41nmt1-Pil1-mCherry-NcNbr1(207-588)(leu1+)*
DY49211	h-	*ura4-D18 ars1::pDUAL-P41nmt1-NcAms1-mECitrine(ura4+) leu1-32::pDUAL-P41nmt1-Pil1-mCherry-NcNbr1(589-867)(leu1+)*
DY49491	h+	*his3-D1 ura4-D18 ura4::pUra4-P41nmt1-mECitrine-CtNbr1(natMX)*

		leu1-32::pDUAL-pDUAL-Pnmt1-Pil1-mCherry-SpAtg8(1-115)(leu1+)
DY49492	h+	*his3-D1 leu1-32 ura4-D18::pUra4-P41nmt1-mECitrine-CtNbr1(natMX)*<br>*ade6-D19::pAde6-P41nmt1-Pil1-mCherry(hphMX)*
DY49493		*his3-D1 leu1-32 SpCpy1-Venus::hphMX*<br>*ura4-D18::pUra4-P41nmt1-CtNbr1-mCherry(natMX)*
DY49494		*his3-D1 leu1-32 atg5Δ::kanMX SpCpy1-Venus::hphMX*<br>*ura4-D18::pUra4-P41nmt1-CtNbr1-mCherry(natMX)*
DY49495		*his3-D1 leu1-32 SpCpy1-Venus::kanMX*<br>*ade6-D19::pAde6-P41nmt1-CtAms1-mCh(hphMX)*
DY49496		*his3-D1 leu1-32 SpCpy1-Venus::kanMX*<br>*ade6-D19::pAde6-P41nmt1-CtAms1-mCh(hphMX)*<br>*ura4-D18::pUra4-P41nmt1-CtNbr1(natMX)*
DY49497		*his3-D1 leu1-32 SpCpy1-Venus::kanMX*<br>*ade6-D19::pAde6-P41nmt1-CtAms1-G2A-mCh(hphMX)*<br>*ura4-D18::pUra4-P41nmt1-CtNbr1(natMX)*
DY49498		*his3-D1 leu1-32 SpCpy1-Venus::kanMX*<br>*ade6-D19::pAde6-P41nmt1-CtAms1-G3A-mCh(hphMX)*<br>*ura4-D18::pUra4-P41nmt1-CtNbr1(natMX)*
DY49499		*his3-D1 leu1-32 atg5Δ::kanMX SpCpy1-Venus::hphMX*<br>*ade6-D19::pAde6-P41nmt1-CtAms1-mCh(hphMX)*<br>*ura4-D18::pUra4-P41nmt1-CtNbr1(natMX)*
DY49500	h+	*his3-D1 leu1-32::pDUAL-pDUAL-Pnmt1-CtAms1-FFH(leu1+)*
DY49501	h+	*his3-D1 leu1-32::pDUAL-pDUAL-Pnmt1-CtAms1-G2A-FFH(leu1+)*

Supplementary Table 2. Plasmids

Name	Description
pDB5132	pDUAL plasmid expressing Pil1-mCherry-CtNbr1 from P41nmt1 promoter
pDB5133	pDUAL plasmid expressing CtAms1-mECitrine from P41nmt1 promoter
pDB5190	pDUAL plasmid expressing CtApe1-mECitrine from P41nmt1 promoter
pDB5176	pDUAL plasmid expressing CtApe2-mECitrine from P41nmt1 promoter
pDB5177	pDUAL plasmid expressing CtApe4-mECitrine from P41nmt1 promoter
pDB5139	pDUAL plasmid expressing Pil1-mCherry-CtNbr1-(635-775) from P41nmt1 promoter
pDB5140	pDUAL plasmid expressing CtAms1-30aa-CtNbr1-FW-13aa-MBP-2x3C-GFP from Pnmt1 promoter
pDB5141	pDUAL plasmid expressing Pil1-mCherry-CtNbr1-(635-775)Y739A from P41nmt1 promoter
pDB5142	pDUAL plasmid expressing Pil1-mCherry-CtNbr1-(635-775)D654A from P41nmt1 promoter
pDB5143	pDUAL plasmid expressing Pil1-mCherry-CtNbr1-(635-775)D658A from P41nmt1 promoter
pDB5144	pDUAL plasmid expressing Pil1-mCherry-CtNbr1-(635-775)R743A from P41nmt1 promoter
pDB5145	pDUAL plasmid expressing Pil1-mCherry-CtNbr1-(635-775)W763A from P41nmt1 promoter
pDB5146	pDUAL plasmid expressing CtAms1-G2A-mECitrine from P41nmt1 promoter
pDB5147	pDUAL plasmid expressing CtAms1-G3A-mECitrine from P41nmt1 promoter
pDB5148	pDUAL plasmid expressing CtAms1-(G2-T5)Δ-mECitrine from P41nmt1 promoter
pDB5149	pDUAL plasmid expressing CtAms1-N14R-mECitrine from P41nmt1 promoter
pDB5150	pDUAL plasmid expressing Pil1-mCherry-CtNbr1-(635-775)D694A from P41nmt1 promoter
pDB5151	pDUAL plasmid expressing Pil1-mCherry-CtNbr1-(635-775)Y695A from P41nmt1 promoter
pDB5152	pDUAL plasmid expressing Pil1-mCherry-CtNbr1-(635-775)R761A from P41nmt1 promoter
pDB5153	pDUAL plasmid expressing Pil1-mCherry-CtNbr1-(635-775)R539A from P41nmt1 promoter
pDB5265	pDUAL plasmid expressing Pil1-mCherry-CtNbr1-(635-775)F651A from P41nmt1 promoter
pDB5213	pDUAL plasmid expressing Pil1-mCherry-CtNbr1(222-588) from P41nmt1 promoter
pDB5214	pDUAL plasmid expressing Pil1-mCherry-CtNbr1(589-883) from P41nmt1 promoter
pDB5215	pDUAL plasmid expressing Pil1-mCherry-CtNbr1(635-775)D654E from P41nmt1 promoter
pDB5216	pDUAL plasmid expressing CtLap2-mECitrine from P41nmt1 promoter
pDB5217	pDUAL plasmid expressing Pil1-mCherry-NcNbr1(207-588) from P41nmt1 promoter
pDB5218	pDUAL plasmid expressing Pil1-mCherry-NcNbr1(589-867) from P41nmt1 promoter
pDB5253	pUra4-natMX plasmid expressing mECitrine-CtNbr1 from P41nmt1 promoter
pDB5226	pAde6-hphMX plasmid expressing Pil1-mCherry from P41nmt1 promoter
pDB5254	pUra4-natMX plasmid expressing CtNbr1-mCherry from P41nmt1 promoter
pDB5255	pUra4-natMX plasmid expressing CtNbr1 from P41nmt1 promoter
pDB5256	pAde6-hphMX plasmid expressing CtAms1-mCherry from P41nmt1 promoter
pDB5257	pAde6-hphMX plasmid expressing CtAms1-G2A-mCherry from P41nmt1 promoter
pDB5258	pAde6-hphMX plasmid expressing CtAms1-G3A-mCherry from P41nmt1 promoter
pDB5259	pDUAL plasmid expressing CtAms1-FFH from Pnmt1 promoter
pDB5260	pDUAL plasmid expressing CtAms1-G2A-FFH from Pnmt1 promoter
pDB5261	pETDuet plasmid expressing His6-GST-CtNbr1(635-775) from T7/lac promoter in *E. coli*
pDB5262	pETDuet plasmid expressing His6-GST-CtNbr1(635-775)D654A from T7/lac promoter in *E.*

	coli
pDB5263	pETDuet plasmid expressing His6-GST-CtNbr1(635-775)W763A from T7/lac promoter in *E. coli*
pDB5264	pETDuet plasmid expressing His6-GST-hNBR1(358-498) from T7/lac promoter in *E. coli*

Supplementary Table 3. Statistics of data collection, structural refinement and model validation

Sample	Ams1-Nbr1 complex
**Data collection**	
EM equipment	FEI Titan Krios
Voltage (kV)	300
Detector	Gatan K2 Summit
Grid	CryoMatrix M024-Au300-R1.2/1.3
Pixel size (Å)	1.04
Electron dose (e^{-}/Å^{2})	50
Defocus range (μm)	1.5-2.0
Number of images	2925
**Map refinement**	
Particles for refinement	692,409
Symmetry	D2
Overall resolution (Å)	2.2
Map-sharpening B factor (Å^{2})	-99.8
**Model composition**	
Protein chains	9
Protein residues	4720
Water	688
**Structural refinement**	
CC_mask	0.80
CC_volume	0.80
CC_peaks	0.57
RMSD of bonds (Å)	0.008
RMSD of angles (°)	0.739
**Validation**	
Molprobity score	2.48
All-atom Clashscore	11.51
Rotamer outliers (%)	0
Ramachandran plot favored (%)	90.72
Ramachandran plot allowed (%)	9.28
Ramachandran plot outliers (%)	0
